# Patient reports of evaluations for interstitial lung disease in patients with connective tissue diseases and rheumatoid arthritis: an international survey

**DOI:** 10.1186/s12931-026-03795-z

**Published:** 2026-07-11

**Authors:** Oliver Distler, Katy Antonopoulou, Ilaria Galetti, Mary J. Wheatley, Rohit Aggarwal, Yannick Allanore, Paul Emery, Yuko Kaneko, Michael Kreuter, Flora Bery-Janusko, Lizette Moros, Anna-Maria Hoffmann-Vold

**Affiliations:** 1 Department of Rheumatology, University Hospital Zurich, University of Zurich, Zurich, Switzerland; 2 AGORA Federation of Associations for People with Rheumatic and Musculoskeletal Diseases (RMDs) of Southern Europe, Athens, Greece; 3 Federation of European Scleroderma Associations (FESCA), Saint Maur, Belgium; 4 National Scleroderma Foundation, Danvers, MA USA; 5 Division of Rheumatology and Clinical Immunology, University of Pittsburgh, Pittsburgh, PA USA; 6 Department of Rheumatology, Université Paris Cité, APHP, Cochin Hospital, Paris, France; 7 NIHR Biomedical Research Centre, Leeds Teaching Hospitals Trust and Leeds Institute of Rheumatic and Musculoskeletal Medicine, University of Leeds, Leeds, UK; 8 Division of Rheumatology, Department of Internal Medicine, Keio University School of Medicine, Tokyo, Japan; 9 Mainz Center for Pulmonary Medicine, Department of Pneumology, Department of Pulmonary, Critical Care & Sleep Medicine, Mainz University Medical Center & Marienhaus Clinic Mainz, Mainz, Germany; 10 Healthcare Affairs & Patient Engagement, Boehringer Ingelheim International GmbH, Ingelheim am Rhein, Germany; 11 Therapeutic Area Inflammation Medicine, Boehringer Ingelheim International GmbH, Ingelheim am Rhein, Germany; 12 Department of Rheumatology, Oslo University Hospital, Postbox 4950 Nydalen, Rikshospitalet, Oslo, 0424 Norway

**Keywords:** Connective tissue diseases, Idiopathic inflammatory myopathies, Interstitial lung disease, Mixed connective tissue diseases, Patient journey, Patient education, Rheumatoid arthritis, Sjögren’s disease, Systemic lupus erythematosus, Systemic sclerosis

## Abstract

**Background:**

Connective tissue diseases (CTDs) and rheumatoid arthritis (RA) share the pulmonary complication of interstitial lung disease (ILD). However, real-world data on patient perspectives and experience regarding screening and evaluations for ILD following CTD/RA diagnosis are limited. We conducted an international, cross-sectional online survey of patients self-reporting as having confirmed CTDs and RA to understand their experience of lung manifestations and ILD screening and/or evaluations.

**Methods:**

Respondents aged ≥18 years with self-reported CTDs/RA with and without ILD were engaged across Africa and Turkey, Europe, Japan and the Americas.

**Results:**

Of 2,225 respondents, 54% were from Europe and 82% were female. Mean ± standard deviation (SD) age was 55 ± 11.6 years. Mean (SD) time from first symptoms to diagnosis with CTD/RA was 14.78 ± 11.37 years. On average, respondents experienced a delay of about 2 years before they were diagnosed with CTD/RA after experiencing their first symptoms. Mean (SD) time from CTD/RA diagnosis to diagnosis with lung conditions was 9.61 ± 10.09 years. Lung screening/evaluations were reported to have been done by 70% of all respondents, mostly as a general or routine status check (39%). Chest high-resolution computed tomography (HRCT) and pulmonary function tests (PFTs) were the most recalled tests to evaluate the lungs for ILD (both 50%), especially among respondents with systemic sclerosis (73% and 87%, respectively). Among respondents who recalled lung evaluation by chest HRCT scan or PFTs, tests were carried out every 1–2 years. Most survey respondents (87%) rated lung screening/evaluations as important.

**Conclusions:**

The survey demonstrated mixed experiences of evaluations for lung manifestations and ILD among patients with CTDs/RA, attributable to differences in the risk of developing ILD and limited clinical guidance for evaluating for ILD in CTDs/RA. Despite their mixed experiences, most respondents showed a positive attitude towards lung evaluations, which could be leveraged to ensure regular, proactive screening and evaluation for ILD to improve patient outcomes. Overall, findings from this survey highlight opportunities to improve physician awareness and standardisation of practice for enhanced patient care.

**Supplementary Information:**

The online version contains supplementary material available at https://doi.org/10.1186/s12931-026-03795-z.

## Background

Connective tissue diseases (CTDs) are a heterogeneous group of mostly rare multiorgan systemic autoimmune diseases that include systemic sclerosis (SSc), Sjögren disease (SjD), mixed connective tissue disease (MCTD), systemic lupus erythematosus (SLE) and inflammatory idiopathic myopathies (IIMs; including dermatomyositis, polymyositis, immune-mediated necrotising myopathy and anti-synthetase syndrome) [1–3]. Rheumatoid arthritis (RA) is a chronic autoimmune inflammatory disease characterised by symmetrical polyarthritis [4]. Interstitial lung diseases (ILDs) are a heterogeneous group of diffuse lung parenchymal disorders [5], and are a common manifestation associated with high morbidity and mortality in many CTDs, including SSc, IIMs and RA [2, 6, 7]. The reported prevalence of ILD varies across CTDs/RA; however, a meta-analysis of published studies including more than 65,000 patients estimated a prevalence of 6% in SLE, 17% in SjD, 41% in IIMs, 47% in SSc, 56% in MCTD, and 11% in RA [8].

Risk factors for the development of CTD-ILD or RA-ILD have been identified, but these conditions may still develop in the absence of established risk factors [3, 9, 10]. Early detection and accurate diagnosis of ILD are important for improved disease management and patient outcomes [9–13], but it would require screening of asymptomatic patients after diagnosis with CTD/RA as substantial lung damage and loss of function may occur before patients report respiratory symptoms [14]. There are variations in current screening practices for ILD among patients with different CTDs/RA [8]. Until recently, no specific screening guidelines existed [3, 15], partly due to the considerable heterogeneity in prevalence, risk factors, disease characteristics, morbidity and mortality across the various CTD-ILD and RA-ILD subtypes [8], which have made it difficult to develop unified guidelines. Prior to 2023, expert consensus statements and clinical guidance for screening and monitoring for ILD only existed for SSc, with recommendations that all patients should undergo screening for ILD upon diagnosis, due to the high risk of developing ILD early in the SSc disease course and the high mortality risk associated with SSc-ILD [10, 13].

Recently, the European Respiratory Society (ERS) and the European Alliance of Associations for Rheumatology (EULAR) developed joint clinical practice guidelines for CTD-ILD/RA-ILD [15]. These guidelines recommend that all patients with SSc and MCTD, and IIM patients with risk factors, should be screened for ILD using chest high-resolution computed tomography (HRCT), and suggest that all patients with RA and SjD with risk factors, and IIM patients without risk factors, could be screened for ILD using chest HRCT [15]. Specific nuances in clinical guidelines for CTD-ILD/RA-ILD may exist between regions. The joint American College of Rheumatology (ACR) and American College of Chest Physicians (CHEST) guidelines for the management of ILD in people with CTDs/RA – also recently developed – conditionally recommend screening any patients with CTD/RA at risk of ILD with pulmonary function tests (PFTs; spirometry, lung volumes and diffusion capacity) and chest HRCT [3]. As the international guidelines to assist physicians in screening for ILD in patients with these diseases are relatively new, patients may experience different journeys, even when diagnosed with similar conditions, depending on how their healthcare professional (HCP) understands the disease [16].

Screening is best carried out through shared discussions and decision-making with patients [3]; the more informed patients are about their disease, the more likely they are to self-advocate for screening. Patients may discuss symptoms that their HCP was not aware of and be referred for further tests, potentially leading to earlier diagnosis [17]. It can also help HCPs when making treatment decisions if they have information about outcomes needing prioritisation (e.g. patients with SSc are willing to trade-off certain side effects for improvement in respiratory symptoms [18]). Disease-specific knowledge, particularly in SSc-ILD, has been described as power by some patients since it gives them more confidence, and makes them feel more in control of their illness [19]. Although patient perceptions and patient-reported outcomes in CTD-ILD have been reported [20], there is a significant unmet information need for real-world data on the current practice of screening and screening methodologies with regards to ILD in CTDs/RA, particularly from the patient’s perspective. The objectives of this survey were to provide insights on patient-reported patterns of screening and evaluation for lung involvement and ILD (including time to diagnosis, type and frequency of evaluations performed) among patients with CTDs/RA, and to describe how these patients understand their care regarding lung involvement.

## Methods

### Study design

The study design for this survey has been described previously [21]. This was an international, cross-sectional online survey of patients self-reporting as having a confirmed diagnosis of SSc, SjD, MCTD, SLE, IIMs and RA. The survey was co-developed by Boehringer Ingelheim in consultation with physician experts (OD, RA, YA, PE, YK, MK and AMHV) and patient experts (patient organisation representatives; KA, IG, MJW and Michael Oeschger) who co-developed and reviewed the survey. Before field work began, the physician and patient experts tested the survey for feasibility and reviewed it for clinical accuracy and relevance to the lived experiences of patients. The survey was made publicly available from 15 September 2023 to 15 March 2024 via an online link hosted by Research Partnership’s (Inizio Advisory) partners.

### Ethics statement

The survey was reviewed by Pearl Institutional Review Board (2023 − 0151, https://www.pearlirb.com/) and received a Category 2 exemption from full review, in accordance with the US Department of Health and Human Services and Code of Federal Regulations (CFR; 45 CFR 46.104(d)(2)) as an online survey with no fixed site, and local institutional review board reviews were not sought. Respondents gave informed consent to participate as part of the survey and could withdraw at any time. The survey complied with all relevant local and regional laws, including the General Data Protection Regulation and good data protection practices outside of the European Union. The survey was conducted according to the principles of the European Pharmaceutical Market Research Association, European Society for Opinion and Marketing Research and the British Healthcare Business Intelligence Association within the European Union, the Insights Association within the United States and the Market Research Society globally, and the respondents’ anonymity was maintained throughout.

### Participant involvement

Apart from Japan, respondents were involved through patient organisations that shared the link to the survey with their members by email, newsletter, websites and social media. To expand reach, the survey link was also shared on the Boehringer Ingelheim corporate social and local media channels, where appropriate and compliant with regulations. Additionally, in some clinical settings, involvement was facilitated through posters and flyers bearing QR codes linking to the survey. In Japan, respondents were involved through a panel provider that invited its members to the survey and compensated them with a small monetary incentive via points with a Japanese online retailer for their time and effort in completing the survey.

### Study population and data collection

Respondents were aged ≥ 18 years and self-reporting a medical diagnosis of one or two CTDs (i.e. SSc, SjD, MCTD, SLE and IIMs [including dermatomyositis, polymyositis, necrotising myopathy or anti-synthetase syndrome]), or RA. To be included in the survey, respondents were required to self-report a diagnosis of their first CTD/RA that had been confirmed by a specialist HCP such as a rheumatologist, internist, pulmonologist or angiologist. Respondents were also required to speak at least one of the 12 pre-specified languages (English, French, German, Italian, Spanish, Portuguese, Greek, Norwegian, Hungarian, Czech, Turkish and Japanese) for survey completion. The survey required respondents to report a diagnosis with lung manifestations or any other conditions, regardless of whether they were related to their CTD/RA. Respondents were considered to present a lung manifestation when they confirmed a diagnosis with ILD or pulmonary arterial hypertension, as well as asthma, emphysema, chronic obstructive pulmonary disease or other lung disease. Respondents reporting a diagnosis with ILD were asked if they were diagnosed with ILD before or after being diagnosed with CTD/RA.

The survey included questions to confirm eligibility, questions relating to the duration of disease, lung manifestations or conditions diagnosed (with priority for ILD if mentioned), recollection of tests performed, awareness of risks and risk factors, sources of information and demographics profile (Additional file 1, Table S1). For the final analysis, complete surveys were preferred; however, incomplete surveys were reviewed on a case-by-case basis to determine whether sufficient data were collected.

## Results

### Survey sample, respondents’ demographics and clinical characteristics

After screening all surveys for completeness and eligibility, 2,225 respondents from Europe (54%; *n* = 1,193), Japan (41%; *n* = 915), Africa and Turkey (3%; *n* = 74) and the Americas (2%; *n* = 43) met the inclusion criteria and were included in the final analysis (Table 1).

The mean ± standard deviation (SD) age of respondents was 55 ± 11.6 years, and 82% were female (Table 1). The primary HCPs who led care for the only or first diagnosis of CTD/RA were predominantly rheumatologists (82% [1,830/2,225]), followed by internists (13% [298 /2,225]), and mainly in hospital (65% [1,455/2,225]) and independent office/clinic (34% [760/2,225]) care settings (Table 1).

**Table 1:** Respondents’ demographics and clinical characteristics by region

Condition, *n* (%)	Total	Region*			
	(*N* = 2,225)	Europe	Japan	Africa and Turkey	Americas
		(n=1,193)	(n=915)	(n=74)	(n=43)
Mean age, years (SD)	55 (11.6)	57 (12.1)	54 (9.6)	44 (14.3)	47 (13.8)
Gender, n (%)					
Female	1,833 (82)	1,084 (91)	644 (70)	64 (86)	41 (95)
Male	382 (17)	105 (9)	265 (29)	10 (14)	2 (5)
Non-binary/prefer not to say	10 (<1)	4 (<1)	6 (1)	0 (0)	0 (0)
Primary HCPs seen, n (%)					
Rheumatologist	1,830 (82)	989 (83)	755 (83)	46 (62)	40 (93)
Internist	298 (13)	147 (12)	121 (13)	28 (38)	2 (5)
Pulmonologist	41 (2)	26 (2)	14 (2)	0 (0)	1 (2)
Dermatologist	21 (1)	8 (1)	13 (1)	0 (0)	0 (0)
Immunologist	25 (1)	17 (1)	8 (1)	0 (0)	0 (0)
Angiologist	10 (<1)	6 (1)	4 (<1)	0 (0)	0 (0)
Care setting usually seen, n (%)					
Hospital	1,455 (65)	816 (68)	591 (65)	40 (54)	8 (19)
Independent office/clinic (medical practice, office, clinic, ambulatory healthcare clinic)	760 (34)	373 (31)	318 (35)	34 (46)	35 (81)
Other	10 (<1)	4 (<1)	6 (1)	0 (0)	0 (0)
Diagnosis					
RA	1,184 (53)	743 (62)	404 (44)	22 (30)	15 (35)
SjD	460 (21)	175 (15)	250 (27)	20 (27)	15 (35)
SSc	376 (17)	289 (24)	54 (6)	23 (31)	10 (23)
SLE	322 (14)	94 (8)	206 (23)	13 (18)	9 (21)
IIM^†^	132 (6)	39 (3)	86 (9)	7 (9)	0 (0)
MCTD	93 (4)	32 (3)	54 (6)	6 (8)	1 (2)
Other autoimmune condition	82 (4)	53 (4)	24 (3)	3 (4)	2 (5)
Lung conditions					
ILD	202 (9)	139 (12)	42 (5)	10 (14)	11 (26)
Asthma	204 (9)	132 (11)	68 (7)	3 (4)	1 (2)
Emphysema or COPD	72 (3)	59 (5)	11 (1)	2 (3)	0 (0)
PAH	52 (2)	34 (3)	8 (1)	7 (9)	3 (7)
Other lung disease	73 (3)	49 (4)	18 (2)	6 (8)	0 (0)
Cardiovascular conditions					
Hypertension	280 (13)	174 (15)	93 (10)	2 (3)	11 (26)
Stroke	31 (1)	16 (1)	13 (1)	1 (1)	1 (2)
Coronary heart disease	28 (1)	15 (1)	10 (1)	2 (3)	1 (2)
Cardiac arrest or heart attack	21 (1)	14 (1)	6 (1)	0 (0)	1 (2)
Other circulatory problem	74 (3)	44 (4)	25 (3)	4 (5)	1 (2)
Renal conditions					
Chronic kidney disease	57 (3)	35 (3)	16 (2)	3 (4)	3 (7)
Other kidney problem	58 (3)	27 (2)	29 (3)	2 (3)	0 (0)
Dermatological conditions					
Skin rashes	128 (6)	70 (6)	46 (5)	8 (11)	4 (9)
Morphoea	21 (1)	14 (1)	5 (1)	2 (3)	0 (0)
Other skin problem	161 (7)	122 (10)	24 (3)	11 (15)	4 (9)
Other conditions					
Raynaud’s phenomenon	460 (21)	275 (23)	159 (17)	15 (20)	11 (26)
Fatigue	255 (11)	198 (17)	36 (4)	14 (19)	7 (16)
Thyroid disease or hypothyroidism	219 (10)	155 (13)	55 (6)	2 (3)	7 (16)
Anaemia	203 (9)	77 (6)	113 (12)	9 (12)	4 (9)
Fibromyalgia	138 (6)	111 (9)	17 (2)	5 (7)	5 (12)
Chronic fatigue syndrome	110 (5)	97 (8)	6 (1)	6 (8)	1 (2)
Dysphagia	64 (3)	55 (5)	6 (1)	1 (1)	2 (5)
Other	74 (3)	47 (4)	22 (2)	4 (5)	1 (2)

*COPD* chronic obstructive pulmonary disease, *HCP* healthcare professional, *IIM* idiopathic inflammatory myopathy, *ILD* interstitial lung disease, *MCTD* mixed connective tissue disease, *PAH* pulmonary arterial hypertension, *RA* rheumatoid arthritis, *SD* standard deviation, *SjD* Sjögren disease, *SLE* systemic lupus erythematosus, *SSc* systemic sclerosis

*Regions: Europe (France, Spain, UK, Norway, Germany, Italy, Switzerland, Czechia, Austria, Portugal, Belgium, Ireland); Africa and Turkey (Algeria, Turkey, Morocco, Tunisia, Mauritius); Americas (US, Canada, Argentina, Chile, Brazil, Ecuador, Mexico, Uruguay, Colombia, Costa Rica, Dominican Republic, Paraguay, Guatemala)

^†^IIM included dermatomyositis, polymyositis, necrotising myopathy and anti-synthetase syndrome

The majority of respondents (91% [2,023/2,225]) were diagnosed with only CTD/RA, and 9% (202/2,225) were diagnosed with CTD-ILD or RA-ILD (Table 1). The most frequently diagnosed conditions were RA (53%) and SjD (21%) (Table 1). In addition to CTDs/RA, respondents had also been diagnosed with various other diseases, including respiratory, cardiovascular, renal, dermatological and other conditions (Additional file 1, Table S2).

### Respondents’ journey until diagnosis of CTD, RA and ILD

**Fig. 1:**
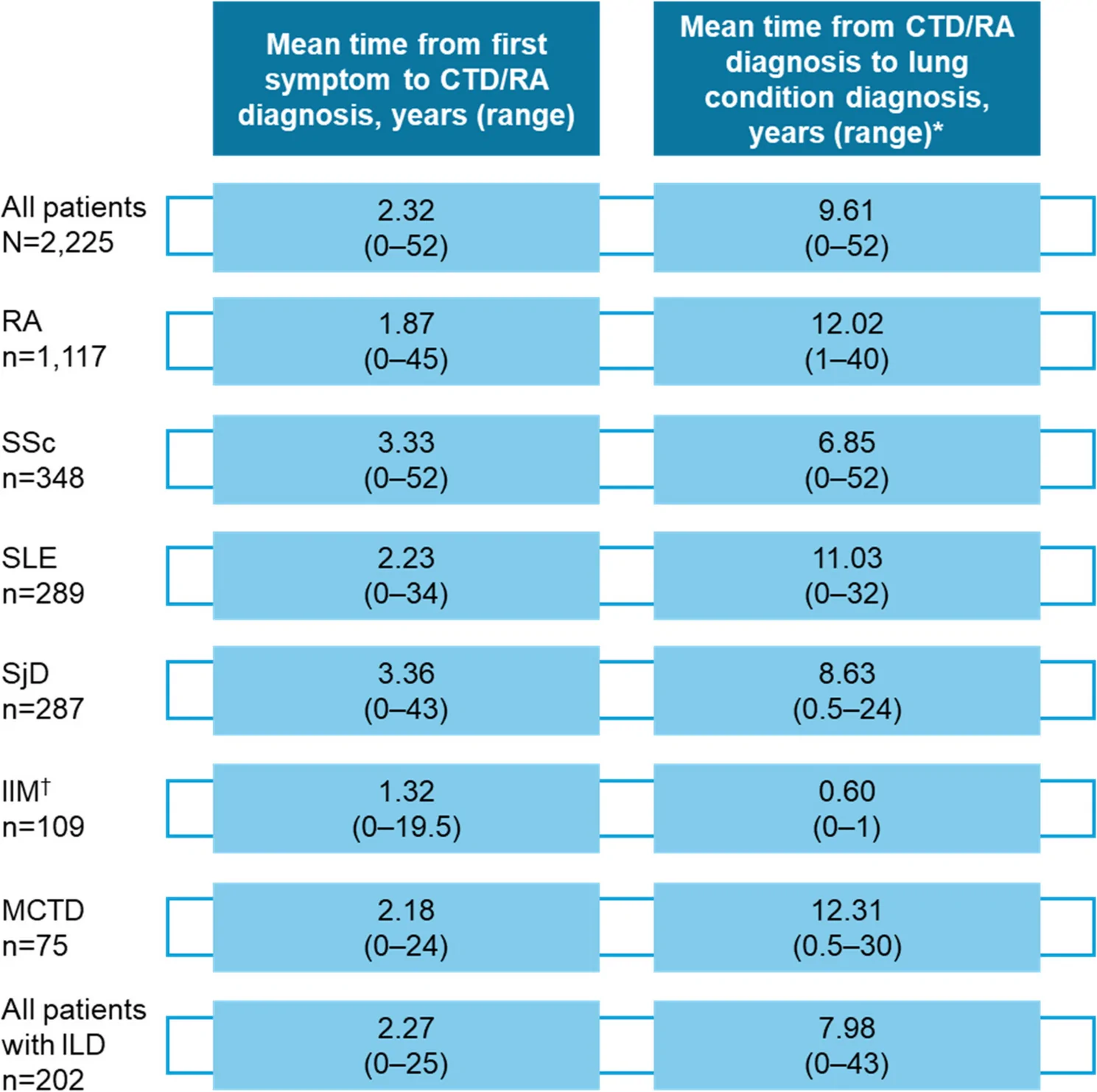
Timeframe for onset of symptoms and CTD, RA and lung condition diagnosis. *Only includes respondents diagnosed with lung condition after CTD diagnosis. ^†^IIM included dermatomyositis, polymyositis, necrotising myopathy or anti-synthetase syndrome. CTD, connective tissue disease; IIM, idiopathic inflammatory myopathy; ILD, interstitial lung disease; MCTD, mixed connective tissue disease; RA, rheumatoid arthritis; SjD, Sjögren disease; SLE, systemic lupus erythematosus; SSc, systemic sclerosis

The mean time since first symptoms of CTD/RA for all respondents was 14.78 ± 11.37 years at survey date (Additional file 1, Figure S1A). On average, respondents experienced a delay of about 2 years before they were diagnosed with CTD/RA after experiencing their first symptoms of the underlying condition. Respondents reported being diagnosed with a lung condition about 9.61 ± 10.09 years following CTD/RA diagnosis (Fig. 1). Among respondents who reported an ILD diagnosis (9% [202/2,225]), just over half (*n* = 111) were diagnosed with ILD after their CTD diagnosis, with a mean time of around 8 years between CTD and ILD diagnoses (Fig. 1); 3% (*n* = 69) were diagnosed with CTD/RA and ILD at the same time, and 1% (*n* = 22) were diagnosed with ILD before CTD/RA. Among respondents who were diagnosed with ILD before CTD/RA, half were diagnosed with CTD/RA later in the same year as their ILD diagnosis. ILD was most frequently reported by respondents with SSc (31% [109/348]), IIMs (22% [24/109] and MCTD (17% [13/75]) (Additional file 1, Table S2).

Lung evaluations were reported to have been done by 1,556/2,225 respondents (70%), and 84% of these (1,303/1,556) were aware of the reasons (Table 2). For most, the evaluation was done as a general or routine status check (510/1,303, 39%) or as an initial check at the beginning of care for their underlying CTD/RA (32% [421/1,303]) (Table 2).

**Table 2:** Lung evaluations in respondents with CTD/RA

Detail	Total	Condition diagnosed first						With ILD	Without ILD
		RA	SSc	SLE	SjD	IIM*	MCTD		
	(*N* = 2,225)	(*n* = 1,117)	(*n* = 348)	(*n* = 289)	(*n* = 287)	(*n* = 109)	(*n* = 75)	(*n* = 202)	(*n* = 2,023)
**Lungs/breathing/lung function evaluations since diagnosis with CTD (e.g. spirometry, imaging, 6-minute walk test), n (%)**									
Lung screening was done, and I know why	1,303 (59)	597 (53)	318 (91)	144 (50)	112 (39)	85 (78)	47 (63)	186 (92)	1,117 (55)
Lung screening was done but I do not know why	253 (11)	141 (13)	13 (4)	46 (16)	37 (13)	8 (7)	8 (11)	9 (4)	244 (12)
Lung screening was not done OR I cannot remember	669 (30)	379 (34)	17 (5)	99 (34)	138 (48)	16 (15)	20 (27)	7 (3)	662 (33)
**Reasons for lung evaluations among those who knew why lungs were evaluated (** ***n*** ** = 1,303), n (%)** ^**†**^									

Detail	**Total**	**RA**	**SSc**	**SLE**	**SjD**	IIM*	**MCTD**	**With ILD**	**Without ILD**
	**(***n*** = 1**,**303)**	**(** *n* * = 597)*	**(** *n* ** = 318)**	**(** *n* ** = 144)**	**(** *n* ** = 112)**	**(** *n* ** = 85)**	**(** *n* ** = 47)**	**(** *n* ** = 186)**	**(***n*** = 1**,**117)**
General/routine lung evaluation	510 (39)	189 (32)	164 (52)	58 (40)	50 (45)	35 (41)	14 (30)	74 (40)	436 (39)
Initial lung evaluation	421 (32)	121 (20)	161 (51)	43 (30)	35 (31)	38 (45)	23 (49)	88 (47)	333 (30)
Lung evaluation before starting new medication	371 (28)	267 (45)	44 (14)	24 (17)	18 (16)	14 (16)	4 (9)	34 (18)	337 (30)
Lung evaluation to check for tuberculosis	219 (17)	160 (27)	15 (5)	21 (15)	9 (8)	9 (11)	5 (11)	13 (7)	206 (18)
Lung evaluation for another reason	297 (23)	156 (26)	37 (12)	47 (33)	28 (25)	22 (26)	7 (15)	41 (22)	256 (23)
**Tests conducted to evaluate the lungs among all respondents whose lungs were evaluated (** ***n*** ** = 1,556), n (%)** ^**†**^									

Detail	**Total**	**RA**	**SSc**	**SLE**	**SjD**	IIM*	**MCTD**	**With ILD**	**Without ILD**
	**(***n = 1*,**556)**	**(** *n* * = 738)*	**(** *n* * = 331)*	**(** *n* * = 190)*	**(** *n* * = 149)*	**(** *n* * = 93)*	**(** *n* * = 55)*	**(** *n* * = 195)*	**(***n = 1*,**361)**
Chest X-ray	1,145 (74)	586 (79)	195 (59)	148 (78)	108 (72)	70 (75)	38 (69)	139 (71)	1,006 (74)
Chest HRCT scan	775 (50)	272 (37)	243 (73)	86 (45)	71 (48)	72 (77)	31 (56)	173 (88)	602 (44)
Pulmonary function test	772 (50)	264 (36)	287 (87)	75 (39)	61 (41)	56 (60)	29 (53)	169 (87)	603 (44)
Auscultation	765 (49)	330 (45)	203 (61)	91 (48)	69 (46)	49 (53)	23 (42)	145 (74)	620 (46)
Oxygen saturation	525 (34)	180 (24)	185 (56)	62 (33)	35 (23)	39 (42)	24 (44)	132 (67)	393 (29)
6-minute walk test	336 (22)	60 (8)	198 (60)	19 (10)	22 (15)	21 (23)	16 (29)	119 (61)	217 (16)
DLco	246 (16)	47 (6)	146 (44)	18 (9)	14 (9)	12 (13)	9 (16)	96 (49)	150 (11)
Lung ultrasound	190 (12)	68 (9)	53 (16)	26 (14)	15 (10)	18 (19)	10 (18)	29 (15)	161 (12)
Other/none of these	65 (4)	25 (2)	12 (4)	10 (5)	11 (7)	7 (8)	0 (0)	11 (6)	54 (4)

*CTD* connective tissue disease, *DLco* diffusing capacity of the lungs for carbon monoxide, *HRCT* high-resolution computed tomography, *IIM* idiopathic inflammatory myopathy, *ILD* interstitial lung disease, *MCTD* mixed connective tissue disease, *RA* rheumatoid arthritis, *SjD* Sjögren disease, *SLE* systemic lupus erythematosus, *SSc* systemic sclerosis

*IIM included dermatomyositis, polymyositis, necrotising myopathy or anti-synthetase syndrome

^†^Respondents could select more than one option. Question was not asked of respondents whose lungs were not evaluated

Almost all respondents with ILD (195/202, 97%) recalled having their lungs evaluated (Table 2). Among the tests used, chest HRCT and PFTs were recalled by 50% (Table 2) and auscultation by 49% (Table 2). Respondents with SSc were more likely than those with other CTDs/RA to recall having certain tests, including chest HRCT scan (73%), PFTs (87%) and auscultation (61%) (Table 2). Respondents with ILD were more likely to recall lung evaluations than those without ILD, including chest HRCT (88% vs. 44%), PFTs (87% vs. 44%) and auscultation (74% vs. 46%) (Table 2). The lung evaluation tests were ordered similarly across HCP specialties, with auscultation the most common test (Table 3). The frequency of lung evaluations, particularly chest HRCT scan and PFTs, was very heterogeneous; most commonly, tests were performed every 1–2 years (Table 4). Among the respondents who recalled a chest HRCT scan or PFT being conducted, 39–40% recalled these being performed every 1–2 years, with higher frequencies reported among respondents diagnosed with SSc (39% for chest HRCT and 53% for PFTs) (Table 4).

**Table 3:** HCP specialties requesting the different lung evaluation tests among respondents whose lungs were evaluated (*n* = 1,556)

Respondents with tests requested by HCP specialty, *n* (%)	HCP specialties										
	Rheumatologist	Pulmonologist	Internist	GP	Cardiologist	Immunologist	Gastroenterologist	Neurologist	Dermatologist	Angiologist	Other
Chest X-ray	698 (45)	157 (10)	92 (6)	111 (7)	24 (2)	14 (1)	2 (<1)	9 (1)	5 (<1)	0 (0)	207 (13)
Auscultation	399 (26)	175 (11)	106 (7)	246 (16)	63 (4)	18 (1)	7 (<1)	4 (<1)	6 (<1)	6 (<1)	89 (6)
Chest HRCT scan	369 (24)	178 (11)	86 (6)	40 (3)	16 (1)	15 (1)	8 (1)	7 (<1)	6 (<1)	2 (<1)	156 (10)
Pulmonary function test	332 (21)	216 (14)	73 (5)	46 (3)	8 (1)	16 (1)	2 (<1)	3 (<1)	10 (<1)	4 (<1)	139 (9)
Oxygen saturation	232 (15)	154 (10)	49 (3)	83 (5)	22 (1)	14 (1)	5 (<1)	1 (<1)	6 (<1)	3 (<1)	83 (5)
6-minute walk test	141 (9)	98 (6)	56 (4)	4 (<1)	12 (1)	10 (<1)	0 (0)	1 (<1)	4 (<1)	1 (<1)	37 (2)
DLco	102 (7)	97 (6)	34 (2)	8 (1)	8 (1)	11 (1)	1 (<1)	0 (0)	1 (<1)	1 (<1)	26 (2)
Lung ultrasound	78 (5)	32 (2)	30 (2)	8 (1)	3 (<1)	5 (<1)	2 (<1)	2 (<1)	1 (<1)	1 (<1)	44 (3)

*DLco* diffusing capacity of the lungs for carbon monoxide, *GP* general or family practitioner, *HCP* healthcare professional, *HRCT* high-resolution computed tomography

**Table 4:** Frequency of tests conducted to assess the lungs among respondents whose lungs were evaluated (*n* = 1,556)

Lung evaluation tests conducted, *n* (%)*	Total	Condition diagnosed first					
		RA	SSc	SLE	SjD	IIM^†^	MCTD
	(*n* = 1,556)	(*n* = 738)	(*n* = 331)	(*n* = 190)	(*n* = 149)	(*n* = 93)	(*n* = 55)
Chest HRCT scan							
Every 3–6 months	104 (7)	26 (4)	41 (12)	8 (4)	8 (5)	16 (17)	5 (9)
Every 1–2 years	302 (19)	91 (12)	128 (39)	24 (13)	23 (15)	26 (28)	10 (18)
Every 3–5 years	108 (7)	44 (6)	34 (10)	11 (6)	8 (5)	7 (8)	4 (7)
Only been done once	208 (13)	94 (13)	31 (9)	26 (14)	29 (19)	20 (22)	8 (15)
Did not know or did not remember	53 (3)	17 (2)	9 (3)	17 (9)	3 (2)	3 (3)	4 (8)
Pulmonary function test							
Every 3–6 months	162 (10)	37 (5)	85 (26)	10 (5)	9 (6)	18 (19)	3 (5)
Every 1–2 years	309 (20)	69 (9)	177 (53)	15 (8)	17 (11)	15 (16)	16 (29)
Every 3–5 years	58 (4)	30 (4)	7 (2)	7 (4)	7 (5)	6 (6)	1 (2)
Only been done once	196 (13)	104 (14)	13 (4)	32 (17)	26 (17)	14 (15)	7 (13)
Did not know or did not remember	47 (3)	24 (3)	5 (2)	11 (6)	2 (1)	3 (3)	2 (4)

*HRCT* high-resolution computed tomography, *IIM* idiopathic inflammatory myopathy, *MCTD* mixed connective tissue disease, *RA* rheumatoid arthritis, *SjD* Sjögren disease, *SLE* systemic lupus erythematosus, *SSc* systemic sclerosis

*Question was not asked of respondents whose lungs were not assessed

^†^IIM included dermatomyositis, polymyositis, necrotising myopathy or anti-synthetase syndrome

### Respondents’ preference regarding evaluation for lung manifestations

**Fig. 2:**
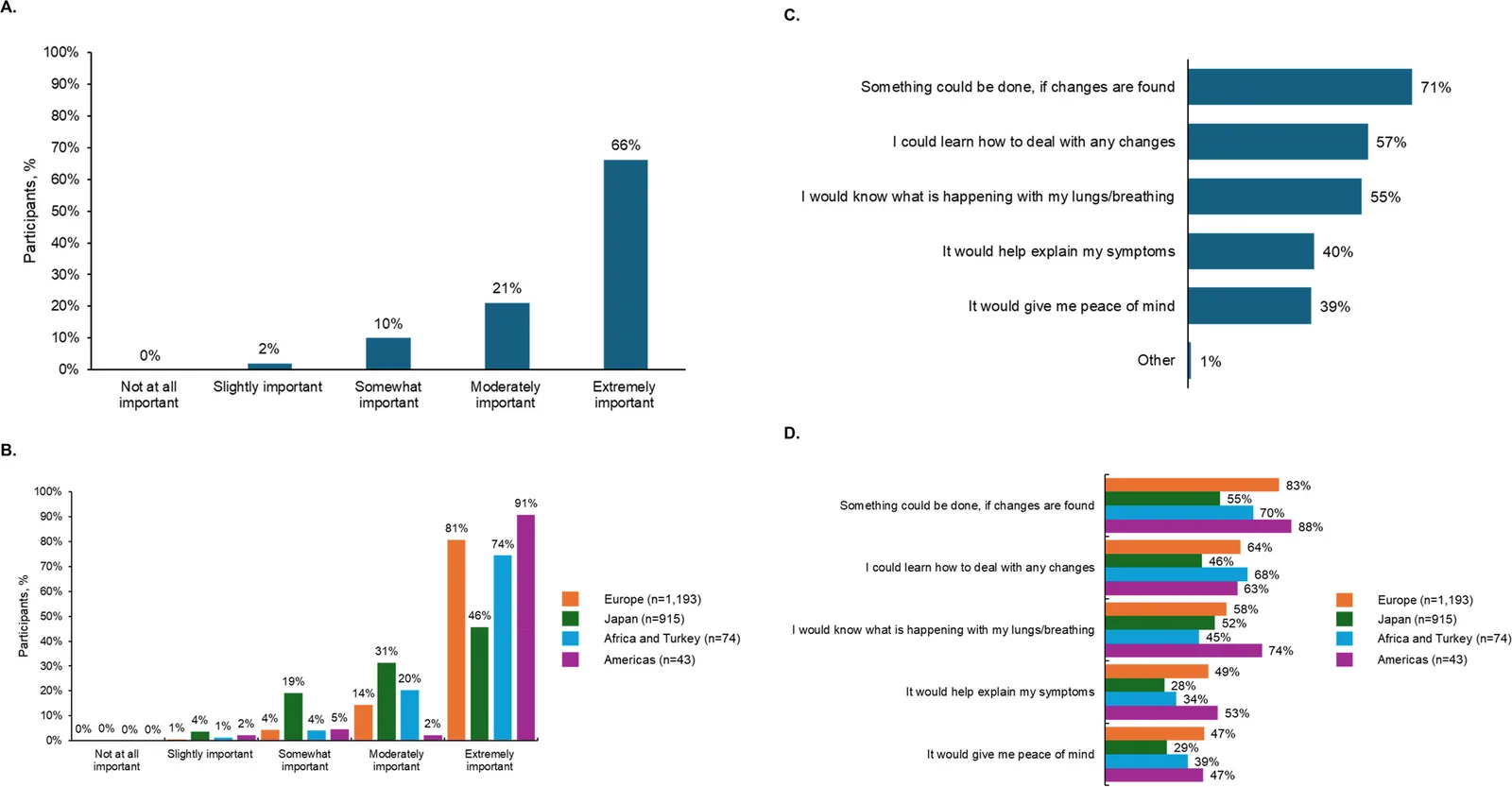
Respondents’ preference regarding lung evaluation. A. Importance of having an HCP evaluate the lungs among all respondents (N=2,225); B. Importance of having an HCP evaluate the lungs stratified by region; C. Reasons for having the lungs evaluated among all respondents; D. Reasons for having the lungs evaluated among all respondents stratified by region. HCP, healthcare professional

When respondents were asked to rank a variety of options that could help with early detection of lung diseases, regular consultation (and/or evaluation) with a pulmonologist or rheumatologist, and knowing the symptoms that should deliver a prompt reaction were considered helpful towards an early detection of lung disease (Additional file 1, Table S3). Across all regions, respondents ranked all screening options to help with early detection of ILD similarly (Additional file 1, Table S4). In Japan, regular evaluation of symptoms by a rheumatologist was ranked first by up to 38% of respondents, while it was ranked second (30%) and third (30%) in Europe (Additional file 1, Table S4). The majority of respondents across all CTDs/RA (87% [1,946/2,225]) scored the importance of an HCP to evaluate the lungs as either ‘extremely important’ (66%) or ‘moderately important’ (21%) (Fig. 2A). Among the regions with the highest number of survey respondents, 81% and 14% of respondents from Europe scored the importance of an HCP evaluating the lungs as ‘extremely important’ and ‘moderately important’, respectively, compared with 46% and 31%, respectively, from Japan (Fig. 2B). For most respondents, regardless of their region (71%; [1,575/2,225]), this was mainly because something could be done if changes were found (Fig. 2C). 

## Discussion

**Fig. 3:**
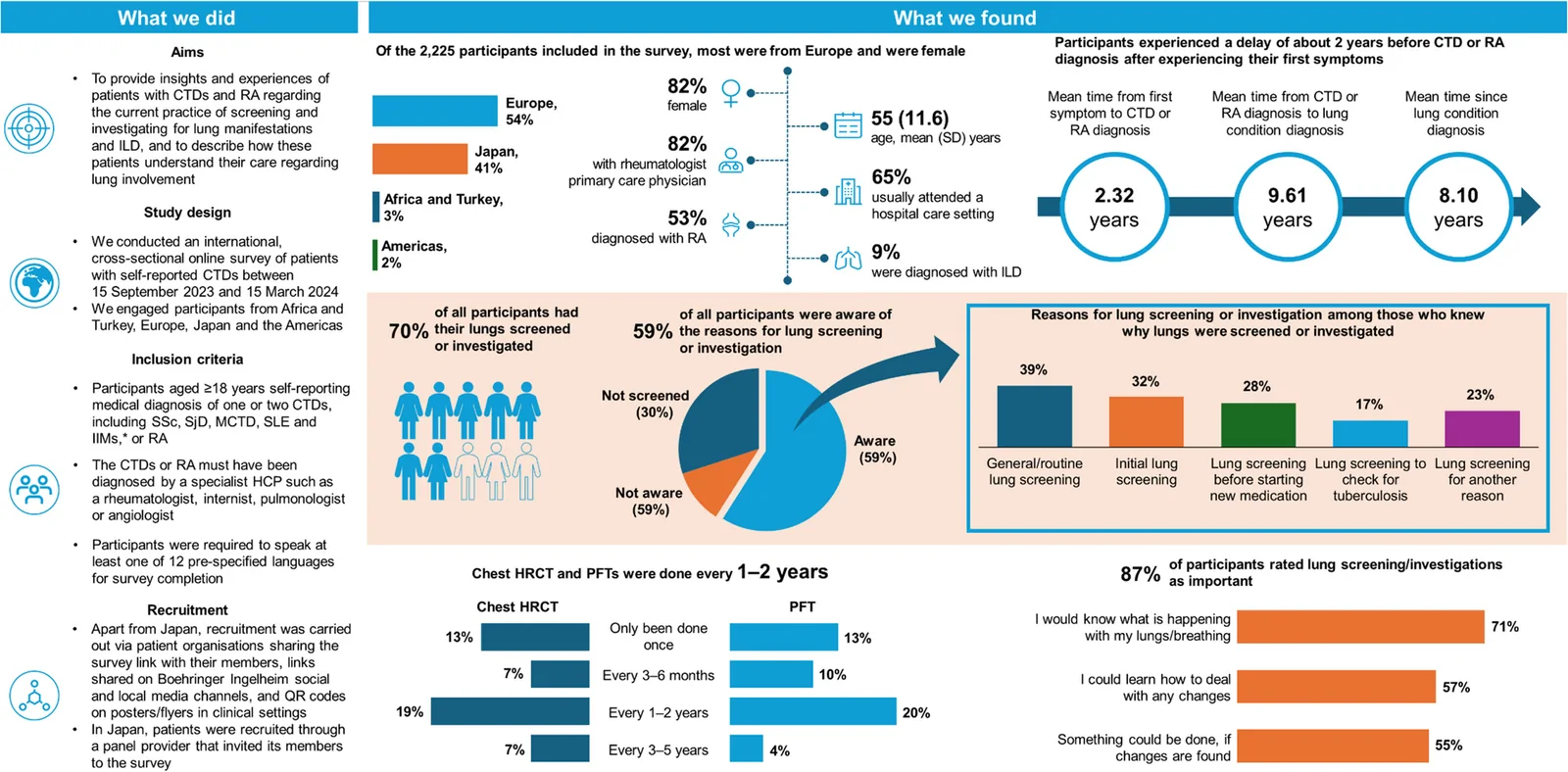
Summary of findings. *IIM included dermatomyositis, polymyositis, necrotising myopathy or anti-synthetase syndrome. CTD, connective tissue disease; HCP, healthcare professional; HRCT, high-resolution computed tomography; IIM, idiopathic inflammatory myopathy; ILD, interstitial lung disease; MCTD, mixed connective tissue disease; PFT, pulmonary function test; RA, rheumatoid arthritis; SD, standard deviation; SjD, Sjögren disease; SLE, systemic lupus erythematosus; SSc, systemic sclerosis

In this survey, we provide patient insights on the current practice of screening and evaluations for ILD in patients with CTDs/RA (Fig. 3). Existing information on patient perspectives and experience regarding screening and evaluations for ILD following CTD/RA diagnosis is limited.

Respondents of this survey reported that they had been living with CTDs/RA for 12 years on average and reported diagnosis with CTD/RA about 2 years after experiencing their first symptoms. Among respondents diagnosed with ILD after CTD/RA, diagnosis was on average 8 years later. ILD was diagnosed prior to CTD/RA in only 1% of the respondents, and among these, about 50% were diagnosed with a CTD/RA later in the same year as their ILD diagnosis. These respondents may have been asymptomatic to the underlying condition, thus delaying diagnosis. This indicates the importance of screening for ILD in patients with CTD/RA, for whom screening is not widely implemented in clinical routine; however, some patients may demonstrate symptoms of ILD months or years before clinical signs of extrapulmonary CTDs/RA appear [22–24], so screening for CTD/RA in patients with ILD is also an important consideration. In retrospective observational studies of patients evaluated in clinics that also focus on internal medicine, the rate of diagnosis with ILD before CTD was reported to range from 8% [25] to 26% [26]. Moreover, timely diagnosis of ILD in CTD/RA may depend on the type of specialist HCP seen and their level of awareness [25, 27, 28]. In our survey, it is unclear which specialist HCPs were involved in the initial diagnosis of ILD. However, half the survey respondents reported that they were diagnosed with RA, which could explain the overall low proportion of patients diagnosed with ILD in the total survey population (9%), as the reported prevalence of ILD in RA in published studies ranges from 6 to 17% [2, 8].

The survey showed that lung evaluation was done for 70% of all respondents, and of these, approximately 4 out of 5 were aware of the reasons, with most recalling that these were done as part of a general or routine assessment. An international survey assessing the attitudes and practices of rheumatologists and pulmonologists caring for patients with RA-ILD showed variability in disease screening and clinical practice among physicians [16]. Similarly, a recent survey of 64 patients with RA-ILD in the UK demonstrated substantial variability in the care that they received, particularly in relation to disease monitoring; only about half of the respondents reported attending respiratory clinics regularly or undergoing regular PFTs [29]. Together with findings from the current survey, there is evidence to advocate for continuous education programmes among HCPs to optimise evaluation practices for patients with CTDs/RA to improve outcomes related to ILD.

Among the recommended tests for screening or diagnosing ILD [3, 5, 10, 13], chest HRCT scans, PFTs and auscultation were recalled as having been performed by almost 1 in every 2 respondents whose lungs were evaluated. However, as respondents could select multiple options, they could have received various combinations of lung tests. When stratified by disease, chest HRCT scans, PFTs and auscultation were more frequently recalled by respondents with SSc compared with all other conditions, possibly reflecting a difference in practice by HCPs regarding the care of patients with SSc [30]. The observation that only approximately half of the respondents recalled undergoing auscultation may reflect recall bias or lack of understanding of the reasons for auscultation, either of which could have influenced how respondents interacted with the survey question. Alternatively, by not conducting auscultation, HCPs could be missing opportunities to detect ILD early, as fine crackles detectable at the lung bases can serve as early clinical clues for ILD [31, 32], which could prompt further investigations with chest HRCT and PFTs.

Due to the high prevalence and mortality risk of SSc-ILD, management guidelines have long recommended screening for ILD in all patients upon diagnosis with SSc [10, 13]. Similarly, although chest HRCT scan and PFTs were frequently recalled as being done every 1–2 years, this was mostly among respondents with SSc. While PFTs are an important diagnostic tool for ILD, they lack sensitivity [33, 34]. Additionally, all other tests (i.e. oxygen saturation, 6-minute walk test, diffusing capacity of the lungs for carbon monoxide and lung ultrasound) that respondents recalled to have received, either lack the diagnostic robustness or sensitivity required to detect ILD, and are not recommended in the latest guidelines for screening and diagnosing ILD [3, 15]. Chest HRCT scans remain the current gold standard for screening and diagnosing ILD [10, 12]. Our findings showed that an elevated proportion of respondents recalled having lung ultrasound performed, possibly due to increasing evidence demonstrating increased sensitivity and specificity to detect ILD [15]. Lung ultrasound can also be performed at the bedside, is low-cost, non-invasive and carries no risk of radiation exposure to the patient [15]. However, lung ultrasound carries a serious risk of bias and indirectness, requires well-trained operators, and the examination and its results are highly dependent on the user; therefore, it can be used as an additional test but chest HRCT must still be performed [15].

SSc was the only condition included in the survey with published screening or monitoring guidelines for ILD at the time of the survey, with ACR/CHEST and ERS/EULAR guidelines for screening and monitoring of ILD in people with other CTDs included in the survey published shortly after the survey concluded in 2024 [3, 15]. Our findings showing high levels of screening and evaluation for ILD in SSc can serve as a blueprint for other CTDs/RA. The 2025 ERS/EULAR clinical practice guideline recommends that all patients with MCTD should be screened for ILD using HRCT; recommends that patients with IIM who have risk factors should be screened for ILD using HRCT (and suggests that patients with IIM without risk factors could also be screened with HRCT, except for those with inclusion body myositis); suggests that patients with RA who have risk factors for developing ILD could be screened using HRCT; and suggests that patients with SjD who have risk factors could be screened for ILD using HRCT [15]. Similarly, the 2023 ACR/CHEST guideline conditionally recommends screening patients with CTDs (i.e. SSc, MCTD, idiopathic inflammatory arthritism, SjD) and RA at risk of developing ILD with chest HRCT over clinical history, physical examination, or PFTs alone [3]. Not screening patients with CTDs/RA who are at risk of developing ILD with HRCT could lead to missing a significant number of ILD cases, yet the risks associated with HRCT, such as radiation exposure, are minimal, and HRCT is generally preferred by patients [15]. No recommendations were provided for patients with SLE nor were any recommendations made regarding the frequency of screening with HRCT in patients with any CTD/RA [3, 15]. Implementation of accelerated programmes to increase awareness among HCPs could drastically improve evaluation practices for ILD in patients with CTDs/RA, leading to an enhanced patient journey.

Respondents had varying opinions regarding evaluation options and HCP specialties that they thought would help best with the early detection of lung disease. Most respondents ranked regular consultation (and/or evaluation) with a pulmonologist or rheumatologist, or a rheumatologist who also focuses on the lungs, and being alerted to symptoms that should prompt reaction, among the best options that could help with early detection of lung disease. Although opinions varied regarding the best HCP specialty, nearly a third of all respondents rated all HCP specialties equally. Interdisciplinary joint consultations between pulmonologists and rheumatologists have been reported as valuable for both the diagnosis and treatment of ILD in CTDs/RA [22]. Due to limited diagnostic guidelines [3, 15] and few approved therapies for ILD, in-depth clinical expertise and a consolidated management plan between pulmonologists and rheumatologists could be pivotal for improving the quality of patient management and outcomes by improving accuracy and confidence in specific CTD-ILD and RA-ILD diagnoses [22].

Almost 9 out of 10 respondents rated the importance of an HCP evaluating the lungs to be ‘extremely important’ or ‘moderately important’; for most, this was primarily because something could be done if changes were found. This result shows patients’ willingness to be proactive with the care they receive. For patients, active involvement in the care that they receive provides a sense of feeling well cared for and being taken seriously [22], and can enhance their participation in shared decision-making regarding their care [29].

### Limitations

Respondents in the survey were self-reporting diagnosed conditions and clinical milestones that were not verified by HCPs, which could have led to inaccurate reporting, as respondents may not have correctly recalled occurrences from several years previously. The survey may have excluded potential respondents as they did not speak one of the qualifying languages, and the online setting could have made it inaccessible for some; only a small fraction of respondents were recruited through posters with a QR code provided at clinics (3%). The majority of respondents were recruited through patient organisations and therefore may have been well informed regarding their disease and actively engaged in their care. Overall, the survey was composed of a sample of convenience, which may not be representative of the general patient population, as no demographic variables apart from age were imposed and only a small number of respondents self-reporting as having confirmed lung involvement were enrolled. There was limited participation of patients from the Americas, and only a small percentage of the respondents included had ILD. The majority of respondents in this survey were from cities and were educated up to post-secondary level, which may not be representative of the overall population of people with CTDs/RA.

## Conclusion

The findings of this survey demonstrate mixed experiences of evaluation for CTD-ILD or RA-ILD among patients, which may be attributable to differences in the risk of developing ILD as well as limited clinical guidance for evaluation for ILD in CTDs/RA. Survey respondents were diagnosed with CTD/RA approximately 15 years after their first symptoms, and were diagnosed with a lung condition almost 10 years following CTD/RA diagnosis. About 70% of all respondents had their lungs evaluated at some point, with chest X-ray, chest HRCT and PFTs among the most recalled tests performed. Despite their mixed experiences, most respondents showed a positive preference towards evaluation for lung manifestations, which could be leveraged by HCPs to ensure that patients are screened or undergo evaluation for ILD regularly. Therefore, integrating routine HCP–patient discussions around the risks of developing ILD and patient education should be prioritised as they could lead to earlier detection and better outcomes. Overall, findings from this survey highlight opportunities to improve physician awareness and standardisation of practice for enhanced patient care. Additionally, both the 2025 ERS/EULAR and 2023 ACR/CHEST guidelines could aid continuous evaluation for ILD in patients with different CTDs/RA once adoption by HCPs gains traction.

## Supplementary Information

Supplementary Material 1.

Supplementary Material 2. Plain-Language Summary Publication https://doi.org/10.60915/Boehringer-figshare.32509641.

## Data Availability

The data that support the findings of this study are not publicly available due to confidentiality and commercial restrictions. The data were collected and analysed by Research Partnership (Inizio Ignite) on behalf of the study sponsor. Requests for access to the data may be directed to the corresponding author; however, any sharing of data is subject to approval by the study sponsor.

## Abbreviations

ACR: American College of Rheumatology
CFR: Code of Federal Regulations
CHEST: American College of Chest Physicians
CTD: Connective tissue disease
ERS: European Respiratory Society
EULAR: European Alliance of Associations for Rheumatology
HCP: Healthcare professional
HRCT: High-resolution computed tomography
IIM: Idiopathic inflammatory myopathy
ILD: Interstitial lung disease
MCTD: Mixed connective tissue disease
PFT: Pulmonary function test
RA: Rheumatoid arthritis
SjD: Sjögren disease
SLE: Systemic lupus erythematosus
SSc: Systemic sclerosis
