# Supplementary material for: Patient reports of evaluations for interstitial lung disease in patients with connective tissue diseases and rheumatoid arthritis: an international survey
Source: Respir Res. 2026 Jul 11;27:383. doi: 10.1186/s12931-026-03795-z (PMC13637092; doi:10.1186/s12931-026-03795-z)
Supplement: Supplementary file 1 [file 12931_2026_3795_MOESM2_ESM.pdf]

July 2026

Data Story

What medical tests are used to check for and monitor interstitial lung disease in patients with connective tissue diseases or rheumatoid arthritis?

Why was this study needed?

- Connective tissue diseases (CTDs) and rheumatoid arthritis (RA) are **autoimmune diseases**
- In patients with CTDs or RA, it is common for the lungs to be targeted by the **immune system**
  - This can affect the lungs, causing lung diseases such as **interstitial lung disease (ILD)** – inflammation or scarring of the tissue between the air sacs in the lungs
  - ILD causes breathing problems and reduces quality of life
- Patients with CTDs or RA should be monitored for signs of ILD, but as international guidelines are quite new, the type of tests chosen and how often they are given by doctors may vary
- More information is needed to understand the current **patient journey** for detection and monitoring of lung complications of CTDs or RA, including ILD, to see where differences exist between regions of the world and where improvements may be made
- This summary describes the results from a survey of patients from different countries to find out how they are checked and monitored for ILD due to CTDs or RA

Connective tissue diseases (CTDs) include:

- Systemic sclerosis
- Sjögren disease
- Mixed connective tissue disease
- Idiopathic inflammatory myopathies (such as polymyositis and dermatomyositis)
- Systemic lupus erythematosus

GLOSSARY

Autoimmune diseases: when the body's defence system (immune system) mistakenly damages healthy parts of the body

Immune system: the body's natural defence system, which can contribute to lung scarring in patients with ILD

Interstitial lung disease (ILD): a group of disorders in which the lungs are inflamed and/or scarred

Patient journey: all interactions a patient has with the healthcare system for a condition, from first noticing symptoms and seeking help, to tests, treatments and ongoing care

How was the study done?

- Medical and patient experts created the survey and checked it was fit-for-purpose before it was made available online
- The survey was available in 12 languages – English, French, German, Italian, Spanish, Portuguese, Greek, Norwegian, Hungarian, Czech, Turkish and Japanese
- The first questions made sure a patient matched the criteria required to take part, before they could complete the rest of the survey
- The remaining questions were to find out:
  - How long they have had a CTD or RA
  - If they had been diagnosed with any lung conditions, including ILD
  - What tests they have had for evaluating the health of their lungs
- Between 15 September 2023 and 15 March 2024, patients with CTDs or RA were invited to complete the online survey via:
  - Emails, newsletters, websites and social media by patient organisations
  - Boehringer Ingelheim social media
  - Posters or flyers containing links to the survey

Who took part in the survey?

- At least 18 years old
- Diagnosed with no more than two CTDs or RA
- Under the care of a specialist doctor*
- Could speak one of the 12 languages the survey was available in

*Rheumatologist, internist, pulmonologist or angiologist

Patients who did not meet these criteria could not take part in this study

2,225 patients took part

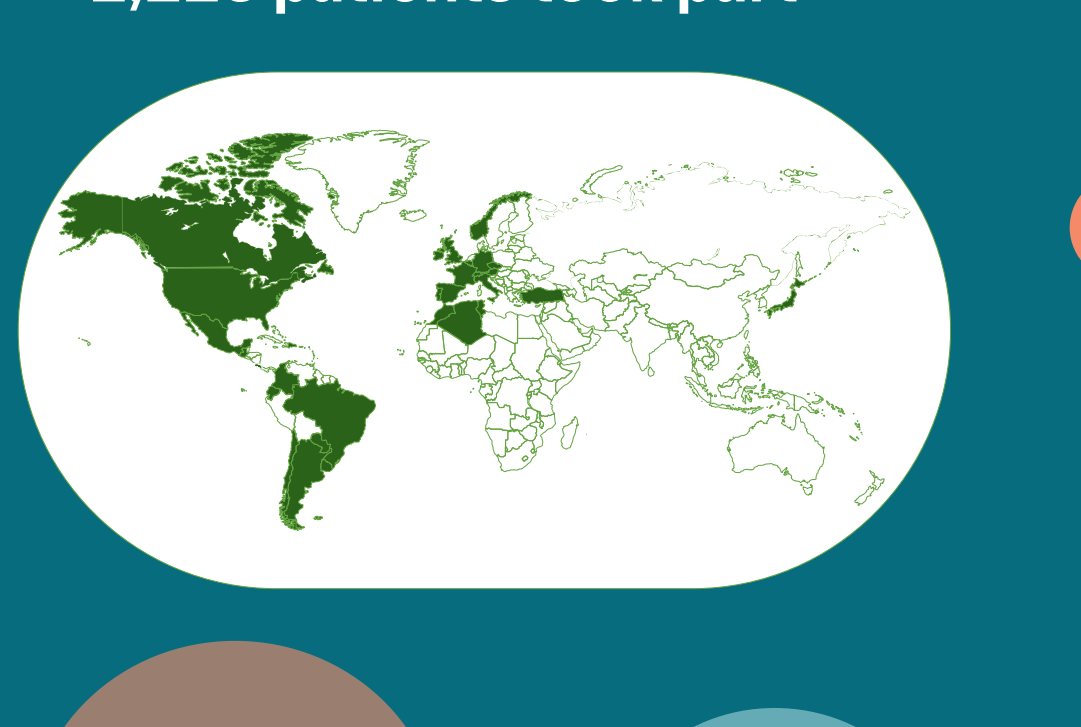

- 3% Africa and Turkey
- 2% North and South America
- 54% Europe
- 41% Japan

82% Were female

55 years Average age

Average length of time since a CTD or RA diagnosis was **10 years**, which ranged from **4 to 19 years**

Result 1: what did the patient journey look like up to diagnosis of a CTD, RA or ILD?

- On average, patients were diagnosed with a CTD or RA around 2 years after experiencing the first symptom
- Most patients surveyed had a CTD or RA diagnosis but did not have an ILD diagnosis, as shown in the image below
  - A lower number of patients were diagnosed with ILD than expected. This may be due to over half being diagnosed with RA, which has a lower chance of ILD developing (around 1 in 10) than seen with most CTDs (between 1 in 2 and 1 in 5). The type of specialist doctor seen and their knowledge of ILD caused by autoimmune disease may also have affected if patients were given an ILD diagnosis, and when
  - Among patients with an ILD diagnosis, most of them (about 95%) were diagnosed with a CTD or RA before, at the same time as or within the same year as their ILD diagnosis

Type of diagnosis

9 out of 10 patients were diagnosed with a CTD or RA only — **91%** A CTD or RA only

1 out of 10 patients were diagnosed with ILD due to a CTD or RA — **9%** A CTD or RA and ILD

Which was diagnosed first?

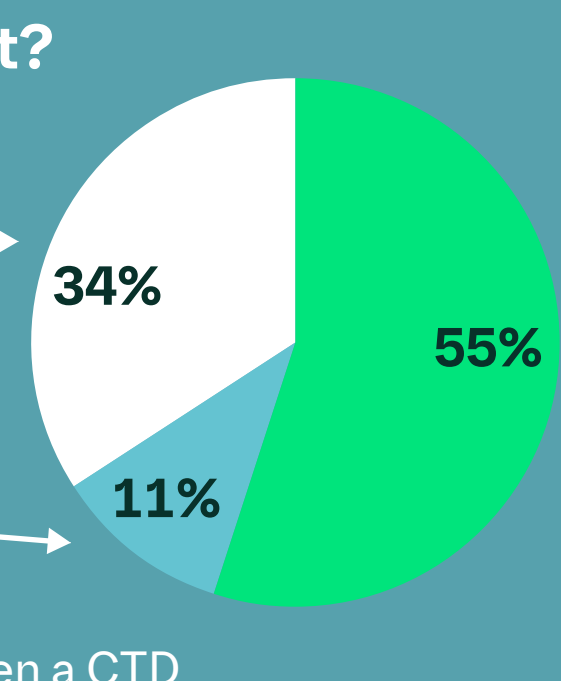

- A CTD or RA and ILD diagnosed at the same time: 34%
- A CTD or RA diagnosed before ILD: 55%
  - Average time between diagnoses was 8 years (A CTD or RA → ILD)
- ILD diagnosed before a CTD or RA: 11%
  - Half of these patients were given a CTD or RA diagnosis in the same year as ILD

Result 2: what did the patient journey look like after diagnosis of a CTD, RA or ILD?

- Lung tests were common (reported as done by 70% of patients), and for most patients, were done as part of routine testing or as a first check after their CTD or RA diagnosis
- Most patients reported they knew the reasons for the lung tests (84%)

How common were lung tests?

7 out of every 10 patients (70%) had lung tests

Of these, most patients (84%) knew why they were having lung tests

Most patients had lung tests as:

- **A first check after a CTD or RA diagnosis (32%)**
- **General or routine checks (39%)**

- The most common types of lung tests done were chest X-ray or computed tomography (CT) scans, lung function tests and listening to the lungs, as shown in the image below

Which tests were done?

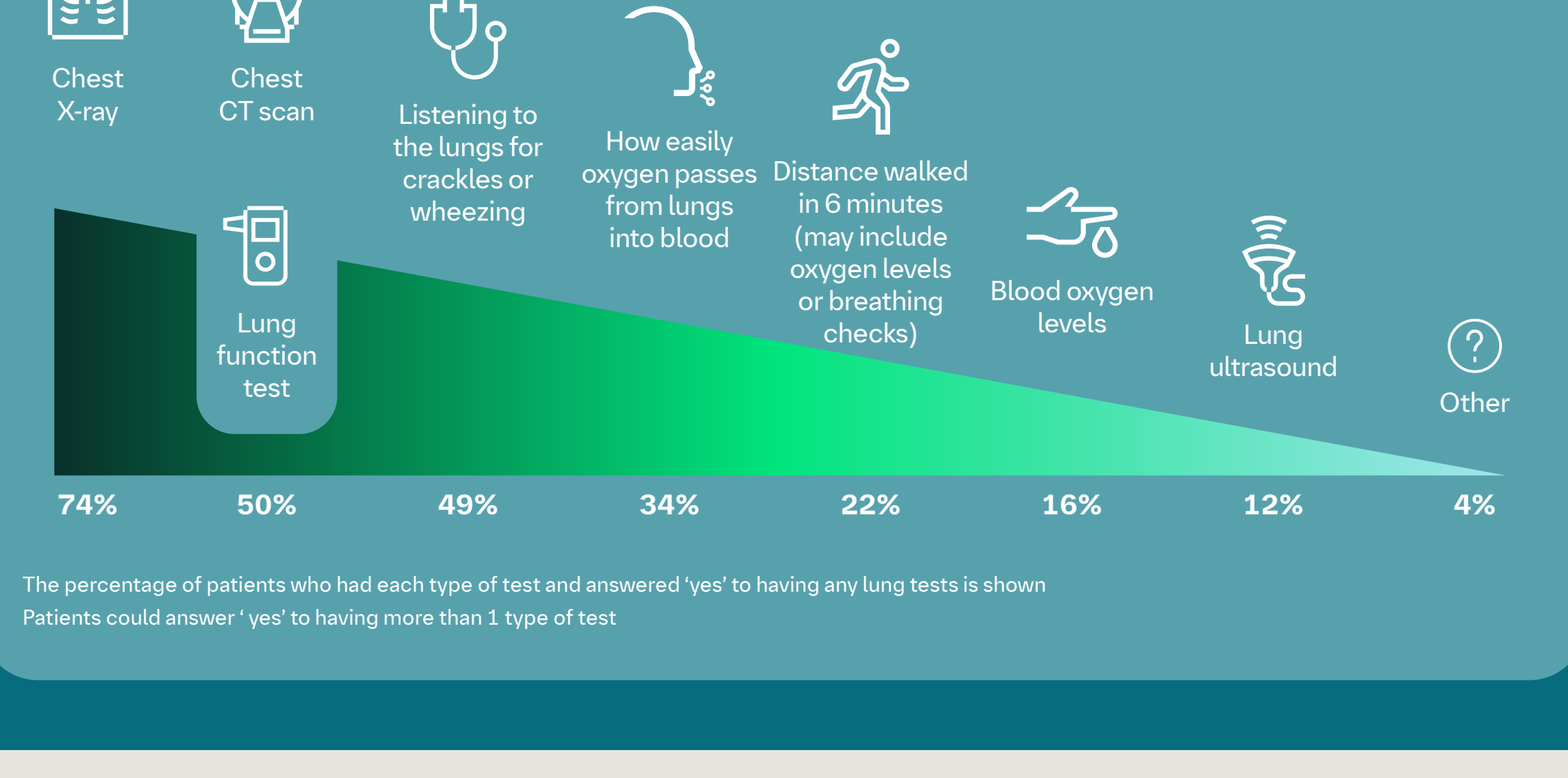

The percentage of patients who had each type of test and answered 'yes' to having any lung tests is shown
Patients could answer 'yes' to having more than 1 type of test

- Similar types of lung tests were ordered by different specialist doctors, such as experts in inflammation of joints, muscles and bones (rheumatologists) and lung specialists (pulmonologists)
- There was no pattern to the number of CT scans or lung tests done per year – most patients had them every 1 to 2 years, as shown in the image below

How often were lung scans or tests done?

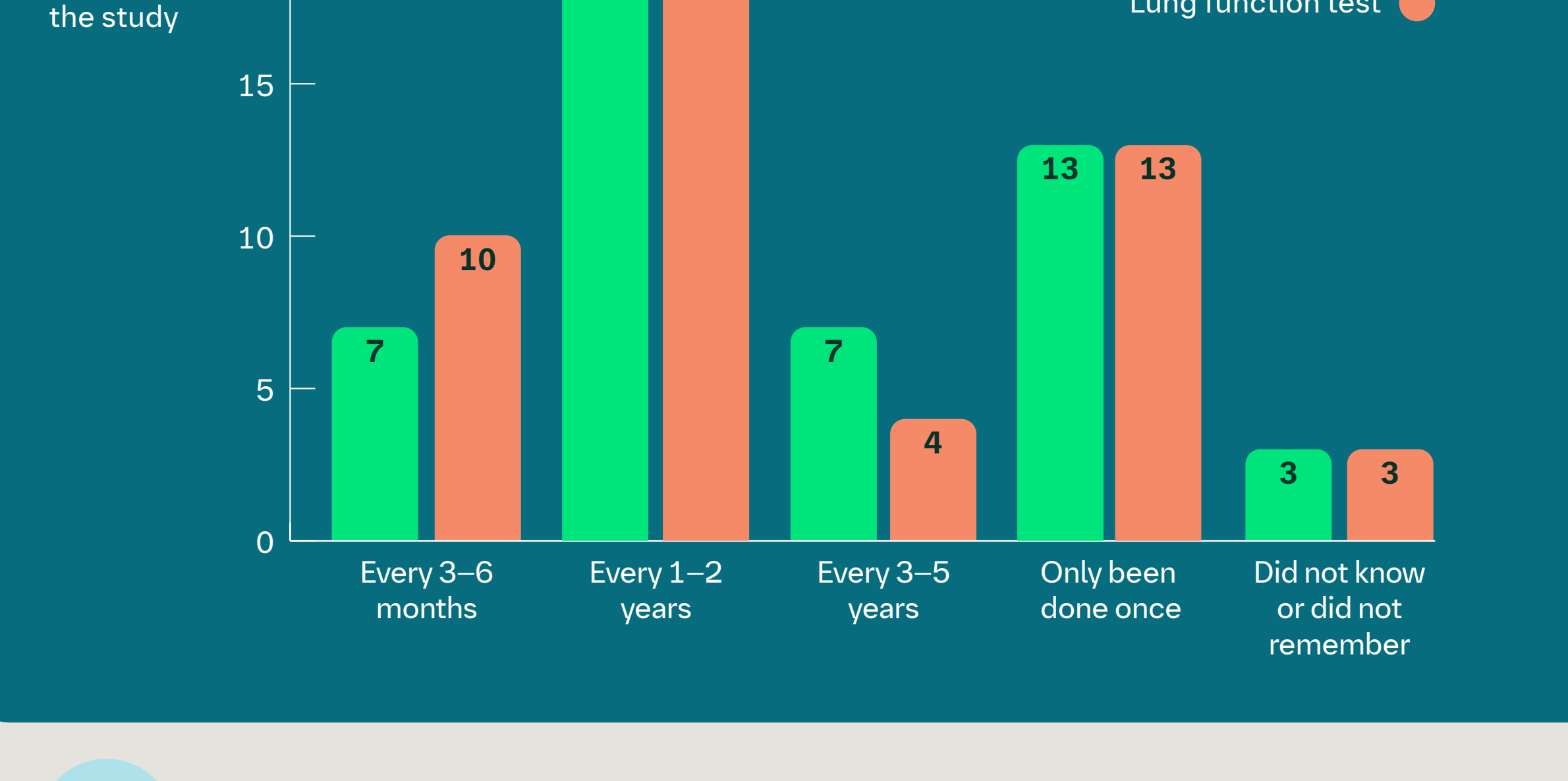

Result 3: how would patients prefer to be monitored for ILD?

- Patients were asked to rate how important they thought monitoring for ILD was, and how they would prefer it to be done
- Most patients (87%) scored the importance of a doctor to check the lungs as either 'extremely important' or 'moderately important'
- Patients across all regions of the world answered that regular appointments (and/or checks and tests) with a pulmonologist or rheumatologist, and knowing the symptoms of ILD that they should tell their doctor about, were considered helpful ways to monitor for ILD
- Most patients (71%) across all regions felt monitoring was important so that something could be done if changes to the lungs were found

What do these results mean?

- Patients with CTDs or RA have different experiences of tests and checks for ILD – for example, the type of lung tests and how often they are done can vary
- This may be due to:
  - Differences in the risk of developing ILD between patients with different types of autoimmune diseases
  - The need for more guidelines for doctors to follow for ILD monitoring of patients with a CTD or RA

What can be done?

- This survey highlights the need to:
  - Make sure doctors are aware of the need to routinely check the lungs of patients with a CTD or RA, so that ILD can be diagnosed as early as possible if it develops
  - Make sure global guidelines for ILD monitoring exist across different autoimmune disease types and are followed by doctors to improve patient care
- Doctors should ensure patients are aware of ILD symptoms to look out for, so patients may pro-actively discuss these with their doctor
- After this survey had been completed, guidelines became available in Europe and the US (called the '2025 ERS/EULAR' and '2023 ACR/CHEST' guidelines). These should be followed by doctors to help monitor for ILD in patients with different CTDs or RA

We are grateful to the patients who took part in this study and to those who support them. Thank you.

This summary is a supplement to the original research publication, which is available at the following link:
Patient reports of evaluations for interstitial lung disease in patients with connective tissue diseases and rheumatoid arthritis: an international survey

Disclosures
This article is intended for patients with connective tissue diseases or rheumatoid arthritis, those that support their care, the general public, and non-specialist healthcare professionals. It has been produced by the authors of the original research publication and reviewed by the scientific community
Writing support was provided by Nicola Winstone, DPhil, of Ashfield Medcomms, an Inizio company, which was funded by Boehringer Ingelheim

SC-CRP-20611
