# Supplementary material for: Patient reports of evaluations for interstitial lung disease in patients with connective tissue diseases and rheumatoid arthritis: an international survey
Source: Respir Res. 2026 Jul 11;27:383. doi: 10.1186/s12931-026-03795-z (PMC13637092; doi:10.1186/s12931-026-03795-z)
Supplement: Supplementary file 2 [file 12931_2026_3795_MOESM1_ESM.docx]

**Additional file 1**

**Patient reports of evaluations for interstitial lung disease in patients with connective tissue diseases and rheumatoid arthritis: An international survey**

Oliver Distler, Katy Antonopoulou, Ilaria Galetti, Mary J. Wheatley, Rohit Aggarwal, Yannick Allanore, Paul Emery, Yuko Kaneko, Michael Kreuter, Flora Bery-Janusko, Lizette Moros, Anna-Maria Hoffmann-Vold

Table S1. Topics included in the survey

Table S2. Respondents’ clinical characteristics by condition diagnosed first

Table S3. Ranking of options to help with early detection of lung disease by condition diagnosed first

Table S4. Ranking of options to help with early detection of lung disease by region

Figure S1. Time since onset of symptoms of CTD/RA and since diagnosis of a lung condition for respondents at the time of the survey

**Table S1. Topics included in the survey**

**Screener**

Chosen language

Country lived in

Source of awareness of research

Source where they first became aware of research

Age

Condition diagnosed with

Type of scleroderma diagnosed with

First CTD diagnosed or RA

First lung condition diagnosed with

Awareness of antibodies in respondent’s blood related to CTD/RA

Lead specialist for their CTD/RA

Other types of physicians they see

**Main survey**

**Duration of disease**

- Where they see the specialist

- Symptoms experienced at the beginning

- First symptom experienced

- First non-Raynaud’s phenomenon symptom experienced for respondents with SSc

- How long ago first symptoms of CTD/RA experienced

- How long ago they were diagnosed with CTD/RA

- **Lung condition diagnosis (ILD prioritised if mentioned, otherwise primary other
  lung condition)**

- Time when diagnosed with lung condition

- How long ago they were diagnosed with lung condition after CTD/RA diagnosis

- Was risk of developing lung manifestations mentioned when diagnosed with CTD/RA diagnosis

- How long ago they were diagnosed with lung condition before CTD/RA diagnosis

- Breathing difficulty as a child

**Screening or evaluation**

- Breathing, lungs, chest or lung function screened or evaluated

- Knowledge of why lungs, breathing or lung function was screened or evaluated

- Reason why lungs, breathing or lung function was screened or evaluated

- Tests carried out

- Frequency of tests

- Type of doctor requesting tests

**Sentiment regarding screening or evaluation**

- Perceived importance of screening or evaluating the lungs

- Reasons why screening or evaluation of the lungs is important/unimportant

- Best option for early detection of lung disease

**Demographics**

- Gender

- Ethnicity (not asked in France)

- Health status

- Perceived health during last week if diagnosed with RA

- Perceived severity of limitation of life choices and activities during last week if diagnosed with SSc

- Perceived health and quality of life if diagnosed with at least one autoimmune condition (not asked for respondents with RA or SSc)

- Highest level of education achieved

- Level of urbanity

- Region

CTD, connective tissue disease; ILD, interstitial lung disease; RA, rheumatoid arthritis; SSc, systemic sclerosis.

**Table S2. Respondents’ clinical characteristics by condition diagnosed first**

| **Condition, n (%)** | **Total** | **Condition diagnosed first** | | | | | |
| --- | --- | --- | --- | --- | --- | --- | --- |
|  |  | **RA** | **SSc** | **SLE** | **SjD** | **IIM*** | **MCTD** |
|  | **(N=2,225)** | **(n=1,117)** | **(n=348)** | **(n=289)** | **(n=287)** | **(n=109)** | **(n=75)** |
| **Diagnosis** | | | | | | | |
| RA | 1184 (53) | 1117 (100) | 19 (5) | 12 (4) | 30 (10) | 3 (3) | 3 (4) |
| SjD | 460 (21) | 92 (8) | 34 (10) | 40 (14) | 287 (100) | 2 (2) | 5 (7) |
| SSc | 376 (17) | 13 (1) | 348 (100) | 2 (1) | 7 (2) | 4 (4) | 2 (3) |
| SLE | 322 (14) | 13 (1) | 1 (0) | 289 (100) | 14 (5) | 1 (1) | 4 (5) |
| IIM | 132 (6) | 6 (1) | 12 (3) | - | 4 (1) | 109 (100) | 1 (1) |
| MCTD | 93 (4) | 10 (1) | 2 (1) | 3 (1) | 3 (1) | - | 75 (100) |
| Other autoimmune condition | 82 (4) | 35 (3) | 13 (4) | 17 (6) | 13 (5) | 3 (3) | 1 (1) |
| **Lung conditions** | | | | | | | |
| ILD | 202 (9) | 36 (3) | 109 (31) | 7 (2) | 13 (5) | 24 (22) | 13 (17) |
| Asthma | 204 (9) | 118 (11) | 17 (5) | 23 (8) | 29 (10) | 14 (13) | 3 (4) |
| Emphysema or COPD | 72 (3) | 43 (4) | 13 (4) | 6 (2) | 5 (2) | 3 (3) | 2 (3) |
| PAH | 52 (2) | 10 (1) | 29 (8) | 4 (1) | 5 (2) | 1 (1) | 3 (4) |
| Other lung disease | 71 (3) | 41 (4) | 9 (3) | 11 (4) | 5 (2) | 7 (6) | - |
| **Cardiovascular conditions** | | | | | | | |
| Hypertension | 280 (13) | 169 (15) | 38 (11) | 23 (8) | 28 (10) | 15 (14) | 7 (9) |
| Stroke | 31 (1) | 14 (1) | 7 (2) | 5 (2) | 4 (1) | - | 1 (1) |
| Coronary heart disease | 28 (1) | 16 (1) | 3 (1) | 5 (2) | 2 (1) | 1 (1) | 1 (1) |
| Cardiac arrest or heart attack | 21 (1) | 12 (1) | 5 (1) | 3 (1) | - | - | 1 (1) |
| Other circulatory problem | 74 (3) | 26 (2) | 12 (3) | 26 (9) | 7 (2) | 1 (1) | 2 (3) |
| **Renal conditions** | | | | | | | |
| Chronic kidney disease | 57 (3) | 20 (2) | 13 (4) | 14 (5) | 5 (2) | 2 (2) | 3 (4) |
| Other kidney problem | 58 (3) | 25 (2) | 5 (1) | 17 (6) | 8 (3) | 2 (2) | 1 (1) |
| **Dermatological conditions** | | | | | | | |
| Skin rashes | 128 (6) | 49 (4) | 16 (5) | 30 (10) | 19 (7) | 5 (5) | 3 (4) |
| Morphoea | 21 (1) | 1 (0) | 15 (4) | 1 (0) | 2 (1) | - | 2 (3) |
| Other skin problem | 161 (7) | 79 (7) | 34 (10) | 21 (7) | 19 (7) | 5 (5) | 3 (4) |
| **Other conditions** | | | | | | | |
| Raynaud’s phenomenon | 460 (21) | 51 (5) | 227 (65) | 78 (27) | 55 (19) | 12 (11) | 37 (49) |
| Fatigue | 255 (11) | 101 (9) | 74 (21) | 25 (9) | 38 (13) | 11 (10) | 6 (8) |
| Thyroid disease or hypothyroidism | 219 (10) | 106 (9) | 38 (11) | 25 (9) | 36 (13) | 9 (8) | 5 (7) |
| Anaemia | 203 (9) | 80 (7) | 32 (9) | 43 (15) | 35 (12) | 8 (7) | 5 (7) |
| Fibromyalgia | 138 (6) | 77 (7) | 17 (5) | 12 (4) | 29 (10) | 1 (1) | 2 (3) |
| Chronic fatigue syndrome | 110 (5) | 57 (5) | 26 (7) | 7 (2) | 16 (6) | 1 (1) | 3 (4) |
| Dysphagia | 64 (3) | 9 (1) | 38 (11) | 5 (2) | 6 (2) | 4 (4) | 2 (3) |
| Other | 74 (3) | 36 (3) | 9 (3) | 12 (4) | 12 (4) | 3 (3) | 2 (3) |

*IIM included dermatomyositis, polymyositis, necrotising myopathy and anti-synthetase syndrome.

COPD, chronic obstructive pulmonary disease; IIM, idiopathic inflammatory myopathy; ILD, interstitial lung disease; MCTD, mixed connective tissue disease; PAH, pulmonary arterial hypertension; RA, rheumatoid arthritis; SjD, Sjögren disease; SLE, systemic lupus erythematosus; SSc, systemic sclerosis.

**Table S3. Ranking of options to help with early detection of lung disease by condition diagnosed first**

| **Options, n (%)** | **Rank** | **Total** | **Condition diagnosed first** | | | | | |
| --- | --- | --- | --- | --- | --- | --- | --- | --- |
|  |  | **(N=2,225)** | **RA** | **SSc** | **SLE** | **SjD** | **IIM*** | **MCTD** |
|  |  |  | **(n=1,117)** | **(n=348)** | **(n=289)** | **(n=287)** | **(n=109)** | **(n=75)** |
| Regular consultation (and/or monitoring) with a pulmonologist | 1st | 754 (34) | 334 (30) | 165 (47) | 82 (28) | 102 (36) | 44 (40) | 27 (36) |
|  | 2nd | 431 (19) | 195 (17) | 80 (23) | 56 (19) | 53 (18) | 28 (26) | 19 (25) |
|  | 3rd | 519 (23) | 271 (24) | 60 (17) | 83 (29) | 63 (22) | 25 (23) | 17 (23) |
|  | 4th | 521 (23) | 317 (28) | 43 (12) | 68 (24) | 69 (24) | 12 (11) | 12 (16) |
| Regular monitoring of symptoms by a rheumatologist | 1st | 561 (25) | 321 (29) | 46 (13) | 85 (29) | 63 (22) | 31 (28) | 15 (20) |
|  | 2nd | 675 (30) | 366 (33) | 84 (24) | 91 (31) | 85 (30) | 27 (25) | 22 (29) |
|  | 3rd | 584 (26) | 264 (24) | 120 (34) | 65 (22) | 85 (30) | 24 (22) | 26 (35) |
|  | 4th | 405 (18) | 166 (15) | 98 (28) | 48 (17) | 54 (19) | 27 (25) | 12 (16) |
| Regular consultation with a rheumatologist who also focuses on the lungs | 1st | 530 (24) | 257 (23) | 76 (22) | 69 (24) | 86 (30) | 23 (21) | 19 (25) |
|  | 2nd | 596 (27) | 280 (25) | 96 (28) | 81 (28) | 91 (32) | 27 (25) | 21 (28) |
|  | 3rd | 579 (26) | 296 (26) | 98 (28) | 76 (26) | 63 (22) | 30 (28) | 16 (21) |
|  | 4th | 520 (23) | 284 (25) | 78 (22) | 63 (22) | 47 (16) | 29 (27) | 19 (25) |
| Alerted to symptoms that should prompt reaction | 1st | 380 (17) | 205 (18) | 61 (18) | 53 (18) | 36 (13) | 11 (10) | 14 (19) |
|  | 2nd | 523 (24) | 276 (25) | 88 (25) | 61 (21) | 58 (20) | 27 (25) | 13 (17) |
|  | 3rd | 543 (24) | 286 (26) | 70 (20) | 65 (22) | 76 (26) | 30 (28) | 16 (21) |
|  | 4th | 779 (35) | 350 (31) | 129 (37) | 110 (38) | 117 (41) | 41 (38) | 32 (43) |

*IIM included dermatomyositis, polymyositis, necrotising myopathy and anti-synthetase syndrome.

IIM, idiopathic inflammatory myopathy; MCTD, mixed connective tissue disease; RA, rheumatoid arthritis; SjD, Sjögren disease; SLE, systemic lupus erythematosus; SSc, systemic sclerosis.

**Table S4. Ranking of options to help with early detection of lung disease by region**

| **Options, n (%)** | **Rank** | **Total** | **Region*** | | | |
| --- | --- | --- | --- | --- | --- | --- |
|  |  |  | **Europe** | **Japan** | **Africa and Turkey** | **Americas** |
|  |  | **(N=2,225)** | **(n=1,193)** | **(n=915)** | **(n=74)** | **(n=43)** |
| Regular consultation (and/or monitoring) with a pulmonologist | 1st | 754 (34) | 476 (40) | 238 (26) | 28 (38) | 12 (28) |
|  | 2nd | 431 (19) | 233 (20) | 170 (19) | 21 (28) | 7 (16) |
|  | 3rd | 519 (23) | 212 (18) | 285 (31) | 10 (14) | 12 (28) |
|  | 4th | 521 (23) | 272 (23) | 222 (24) | 15 (20) | 12 (28) |
| Regular monitoring of symptoms by a rheumatologist | 1st | 561 (25) | 201 (17) | 345 (38) | 9 (12) | 6 (14) |
|  | 2nd | 675 (30) | 352 (30) | 290 (32) | 19 (26) | 14 (33) |
|  | 3rd | 584 (26) | 357 (30) | 189 (21) | 28 (38) | 10 (23) |
|  | 4th | 405 (18) | 283 (24) | 91 (10) | 18 (24) | 13 (30) |
| Regular consultation with a rheumatologist who also focuses on the lungs | 1st | 530 (24) | 272 (23) | 227 (25) | 18 (24) | 13 (30) |
|  | 2nd | 596 (27) | 284 (24) | 284 (31) | 17 (23) | 11 (26) |
|  | 3rd | 579 (26) | 341 (29) | 205 (22) | 20 (27) | 13 (30) |
|  | 4th | 520 (23) | 296 (25) | 199 (22) | 19 (26) | 6 (14) |
| Alerted to symptoms that should prompt reaction | 1st | 380 (17) | 244 (20) | 105 (11) | 19 (26) | 12 (28) |
|  | 2nd | 523 (24) | 324 (27) | 171 (19) | 17 (23) | 11 (26) |
|  | 3rd | 543 (24) | 283 (24) | 236 (26) | 16 (22) | 8 (19) |
|  | 4th | 779 (35) | 342 (29) | 403 (44) | 22 (30) | 12 (28) |

*Regions: Europe (France, Spain, UK, Norway, Germany, Italy, Switzerland, Czechia, Austria, Portugal, Belgium, Ireland); Africa and Turkey (Algeria, Turkey, Morocco, Tunisia, Mauritius); Americas (US, Canada, Argentina, Chile, Brazil, Ecuador, Mexico, Uruguay, Colombia, Costa Rica, Dominican Republic, Paraguay, Guatemala).

**Figure S1. Time since onset of symptoms of CTD/RA and since diagnosis of a lung condition for respondents at the time of the survey**

**A. Time since first symptom of CTD/RA; B. Time since lung condition diagnosis***

**A.**

**B.**

*Lung conditions diagnosed were categorised as asthma, emphysema or COPD, ILD, PAH and other lung diseases.

^†^IIM included dermatomyositis, polymyositis, necrotising myopathy or anti-synthetase syndrome.

^‡^Among respondents diagnosed with ILD after CTD (n=111), about 18% were diagnosed with ILD later in the same year as CTD diagnosis.

**^§^**Among respondents diagnosed with ILD before CTD (n=20), 50% were diagnosed with ILD earlier in the same year as CTD diagnosis.

COPD, chronic obstructive pulmonary disease; CTD, connective tissue disease; IIM, idiopathic inflammatory myopathy; ILD, interstitial lung disease; MCTD, mixed connective tissue disease; PAH, pulmonary arterial hypertension; RA, rheumatoid arthritis; SD, standard deviation; SjD, Sjögren disease; SLE, systemic lupus erythematosus; SSc, systemic sclerosis.
